# Plastic-Degrading Microbial Consortia from a Wastewater Treatment Plant

**DOI:** 10.3390/ijms252312747

**Published:** 2024-11-27

**Authors:** Andrea Salini, Luca Zuliani, Paolo Matteo Gonnelli, Marco Orlando, Andrea Odoardo, Daniele Ragno, Martina Aulitto, Claudio Zaccone, Salvatore Fusco

**Affiliations:** 1 Biochemistry and Industrial Biotechnology (BIB) Laboratory, Department of Biotechnology, University of Verona, 37134 Verona, Italy; andrea.salini@univr.it (A.S.);; 2 Department of Biotechnology and Biosciences, University of Milano Bicocca, 20126 Milano, Italy; marco.orlando@unimib.it; 3 Department of Chemical, Pharmaceutical and Agricultural Sciences, University of Ferrara, 44121 Ferrara, Italydaniele.ragno@unife.it (D.R.); 4 Department of Biology, University of Naples Federico II, 80126 Naples, Italy; martina.aulitto@unina.it; 5 Lab of Soil and Biomass Chemistry, Department of Biotechnology, University of Verona, 37134 Verona, Italy; claudio.zaccone@univr.it

**Keywords:** polylactic acid (PLA), polyethylene terephthalate (PET), polyesters, wastewater treatment plant (WWTP), microbial plastic degradation, thermophilic enzymes, biocatalytic recycling, plastic-active enzymes, activated sludge

## Abstract

Plastic waste pollution has become a global crisis, with millions of tons of plastic expected to accumulate in landfills and in natural environments, posing a serious threat to wildlife and human health. As current recycling methods remain inefficient, there is an urgent need for innovative enzymatic solutions to break down plastics and enable a circular economy approach. In this study, we explore the plastic-degrading potential of microorganisms enriched from activated sludge (AS) sourced from a municipal wastewater treatment plant (WWTP)—a known microplastic-contaminated industrial niche. Five microbial consortia (i.e., microbiomes) were enriched under selective pressure using low-carbon conditions and high concentrations of polyester polymers, including post-consumer PET, post-consumer PLA, and virgin PLA. Enrichment was performed for 100 days at 37 °C and 50 °C, followed by microbiomes isolation and metagenomic analysis to identify plastic-active bacteria and their enzymes. The results revealed that PLA polymers, but not post-consumer PET, were effectively degraded by the microbiomes, as confirmed by nuclear magnetic resonance (NMR) and gel permeation chromatography (GPC), showing significant molecular weight reduction compared to the abiotic controls. Microbial community analysis highlighted a distinct enrichment profile driven by the polymer composition and the temperature. At 50 °C, the *Bacillales* order became the predominant population, whereas at 37 °C, a more diverse community within the *Proteobacteria* and *Actinobacteria* phyla were selected. Nonetheless, the enriched microbial communities at both temperatures included phyla with members known for polyester degradation. Moreover, at 50 °C, enrichment of putative PET/PLA hydrolases was also observed. These findings suggest that AS microorganisms are a reservoir of polyester-active enzymes, particularly PLA-depolymerases, and hold promise for advancing biotechnological strategies to mitigate plastic pollution through re- and up-cycling.

## 1. Introduction

Petroleum-based plastics are employed in the manufacture of a multitude of consumer goods and have become essential materials in our everyday lives. Global plastic production has escalated significantly in the last few decades; indeed, world plastic production has nearly doubled since the 2000s, reaching a total of 400 million tons in 2022 [1,2]. This unprecedented production rate is driven by the demand for durable, lightweight, and versatile materials that are useful in a variety of industrial sectors, including packaging, textiles, automotive, electronics, and construction.

Nevertheless, despite implementing new policies for the utilisation and disposal of plastic products, the plastic supply chain has yet to achieve a circular model [3]. It is estimated that, as of 2019, less than 10% of all plastic waste was recycled, with the majority being either landfilled (50%) or incinerated (19%) [1]. The remaining 20% comprises mismanaged plastics, which accumulate in natural habitats, including marine, terrestrial and freshwater ecosystems [4,5,6]. Mismanaged plastic waste undergoes gradual degradation processes resulting in the formation of microplastics (size ranges from 1 µm up to 5 mm [7]) due to biological and physicochemical factors, such as UV irradiation, temperature, oxygen, and mechanical forces [8]. Microplastics can be ingested by various organisms, allowing them to enter the trophic chain and accumulate in body tissues. Studies have found microplastics in the intestines of terrestrial animals, freshwater fauna, and marine organisms, highlighting their widespread presence and potential for long-term ecotoxicological harm [9]. Moreover, microplastics act as carriers for environmental pollutants by leaking plasticizers (e.g., phthalates), stabilizing additives (e.g., organotin compounds, alkylphenols), and colouring agents [10]. In addition, they absorb and transfer organic and inorganic pollutants, such as polychlorinated biphenyls, heavy metals, antibiotics and perfluorinated compounds [9]. As microplastics migrate through ecosystems, they facilitate the biomagnification of these pollutants up the food chain, representing a threat not only to wildlife but also to human health. The considerable stability of plastic polymers in the environment, with estimated half-lives ranging from dozens to thousands of years [11], and their incorporation into geological strata, have led to the conclusion that plastic is a hallmark of the Anthropocene [12]. This has led to the coining of the term ‘Plasticene’ to refer to the current era [13]. As plastic production is projected to triple by 2060 [14], there is a compelling need for alternative responses to the current waste management system. For instance, the use of biocatalysts can represent an efficient alternative to traditional mechanical and chemical recycling [15].

Plastic polymers differ greatly in terms of biodegradability because of their different physicochemical properties (e.g., chemical composition, hydrophobicity, crystallinity and particle size) and environmental conditions (e.g., light, oxygen, and temperature) [11,16]. Indeed, environmental conditions play a critical role in initiating plastic weathering and selecting the plastisphere microbial community that colonizes the polymer surface. Chemical degradation of plastics involves, at first, hydrolysis or oxidation depending on the chemical composition of the polymer. Polyolefins, including polyethylene (PE), polystyrene (PS), polypropylene (PP) and polyvinyl chloride (PVC), possess a highly stable C-C backbone that requires oxidation, either abiotic or enzymatic, prior to sequential hydrolytic depolymerisation. The presence of aromatic groups and manufacturing impurities can act as chromophores, allowing the formation of radicals upon exposure to ultraviolet radiation. This results in the formation of free radicals, which can then give rise to a series of chemical reactions, including chain scission, oxidation, or cross-linking [8]. Recently, a few enzymes, belonging to the phenol oxidase family, have been characterised for their ability to oxidise the aliphatic backbone of PE. These include two laccases that were isolated from *Rhodococcus opacus* R7 [17].

In contrast, plastics that contain hydrolysable bonds, such as esters or amides, are more susceptible to enzymatic degradation. Indeed, polyesters, such as polyethylene terephthalate (PET)—a petroleum-based plastic consisting of repetitive units of terephthalic acid (TPA) and ethylene glycol (EG)—and polylactic acid (PLA)—a bioplastic composed of D- and/or L-lactic acid (LA)—have been the object of several studies over the past decade [18]. Research has aimed to understand their susceptibility to enzymatic degradation and the potential for their valorisation. These materials can be hydrolysed to yield defined monomers (i.e., TPA, EG, and LA), which can then be used to manufacture new virgin plastics with the same properties as the starting material or repurposed to higher-value products through upcycling [19]. To date, the plastic-active enzyme database (PAZy) [20] includes more than a hundred biochemically characterized PET hydrolytic enzymes (PHEs), and 38 PLA-depolymerases. PHEs belong mainly to the carboxylic ester hydrolase class (EC 3.1.1.-), specifically comprising carboxylesterases, cutinases, and lipases [18]. Of these, cutinases have been demonstrated to exhibit the most effective hydrolytic activity toward PET due to their ability to work in thermophilic conditions [21]. Cutinases are also effective on PET, as their natural substrate is a hydrophobic polyester, containing aromatic moieties. Similarly, PLA-depolymerases belong to the hydrolase class, which exhibit activity on the ester bond (i.e., PHEs, EC 3.1.1.-) and the peptide bond (i.e., proteases, EC 3.4.-). PLA-degrading proteases, such as Proteinase K [22], specifically target poly(L-lactide) (PLLA), due to the structural similarities between PLLA and their natural substrate, namely silk fibroin, which is mostly composed of L-alanine or glycine units [23]. Conversely, lipases and cutinases prefer poly(D-L-lactide) (PDLLA), but they do not show a strict enantioselectivity towards the substrate [24].

It is important to note that plastic-active enzymes are extracellular and are secreted after the colonization of the plastic surface by the microbial community. The formation of biofilms causes initial biodeterioration, which is followed by polymer biofragmentation due to the secretion of extracellular oxidases and hydrolases. The released oligomers and monomers are then assimilated into the microbial cells, where they undergo further catabolism and may ultimately result in mineralisation [25]. The existing research indicates that the microbial community within the plastisphere differs significantly from that of the surrounding environment, exhibiting lower species richness but greater evenness. Bacterial taxa enriched on plastic surfaces in both marine and terrestrial environments are predominantly *Proteobacteria* and *Actinobacteria*, which are also the phyla that comprise characterized members with the most promising plastic-degradation capabilities [26,27]. In laboratory conditions, the enrichment strategy has been extensively exploited using diverse inoculum matrices, including those derived from landfills, sewage wastewater, anaerobic digesters, and mangroves [28,29,30,31]. For instance, *Ideonella sakaiensis*, which was isolated from a PET bottle recycling site, has demonstrated the ability to degrade and assimilate PET at mesophilic temperatures, using two enzymes, IsPETase and IsMHETase [32]. Similarly, the utilization of artificial microbial consortia has been investigated for the degradation of plastic waste, exploiting the superior metabolic capabilities of a diverse microbial community in comparison to a single strain [33]. This methodology represents a more promising possibility for the effective remediation of mixed plastic waste.

In this study, the plastic-degrading potential of microbes enriched from the activated sludge (AS) collected from a municipal wastewater treatment plant (WWTP) was investigated. WWTPs are industrial niches that are heavily contaminated with microplastics. They are a significant source of microplastic contamination in both aquatic and terrestrial ecosystems [1]. This is due to the incomplete decontamination of inlet wastewater and the application of contaminated AS as a soil amendment. In a study conducted by Magni et al. [34] on the largest WWTP in Northern Italy, it was found that 83% of the incoming microplastic fibres were polyesters, likely originating from clothes washing. Therefore, the microorganisms in AS are consistently exposed to plastic polymers and may represent a significant reservoir of polyester-active enzymes. Enrichment cultures were established, applying selective pressure through low carbon availability and high concentrations of three different polyester polymers: post-consumer PET from water bottles (PET), post-consumer PLA from cups (cPLA), and virgin 3D-printing PLA granules (gPLA). After 100 days of cultivation, the enriched microbial consortia (i.e., microbiomes) were isolated, and their DNA was sequenced to assess the presence of plastic-active bacteria and enzymes. PLA degradation during the enrichment process was confirmed using different techniques, including nuclear magnetic resonance (NMR), elemental (CHNS) and thermal (TGA-DSC) analyses, and its degree of molecular weight reduction was determined via gel permeation chromatography (GPC). Metagenomic analysis showed that temperature had a significant impact on microbial selection, with *Bacillales* dominating in both microbiomes enriched at 50 °C along with an increase in abundance of genes encoding for putative PLA-degrading enzymes. At 37 °C, a more diverse microbial community was enriched. However, this community, despite being able to degrade PLA, did not show an enrichment of PLA depolymerases like those previously characterized. These findings confirm that activated sludge sourced from WWTPs can be a valuable resource for polyester degradation.

## 2. Results

### 2.1. Enrichment and Isolation of Microbial Consortia

The AS from a local municipal WWTP was used as the inoculum of enrichment cultures to select for plastic-degrading microbial consortia (i.e., microbiomes) able to thrive on post-consumer and virgin plastic polymers. Two temperatures were chosen (i.e., 37 and 50 °C) to select for mesophilic and moderate thermophilic microbiomes. The temperature of 37 °C reflects mesophilic conditions, consistent with the operational temperature of municipal WWTPs. In contrast, 50 °C was chosen as it is closer to the glass transition temperature of both polymers, thus increasing the efficiency of enzymatic degradation. At the beginning of the cultivation, colony forming unit (CFU) counting and optical density (OD_600nm_) measurements were carried out to assess microbial proliferation after the spent medium exchanges performed every 10 days (i.e., 10, 20 and 30 days post-inoculation). CFU counting was performed during the first phase of the enrichment (i.e., 0–20 days) due to the initial dark colour of the cultures (Figure S1A) leading to the impossibility of monitoring microbial growth by turbidity measurements at 600 nm.

**Figure 1:**
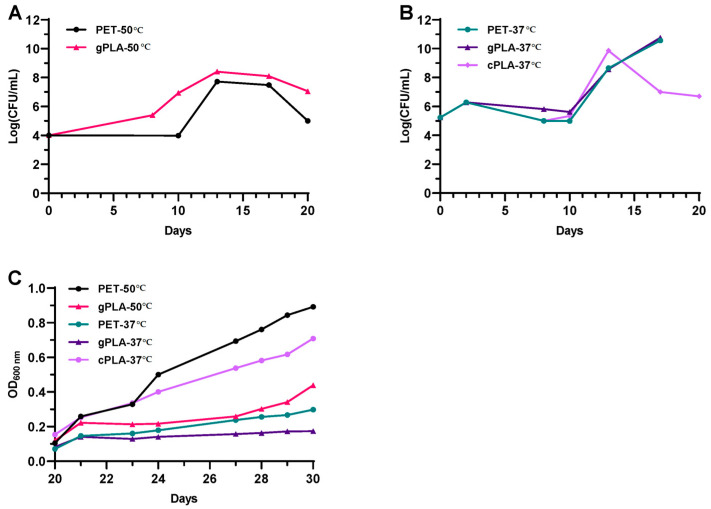
Microbial biomass measurements. (**A**) CFU counting of cultures enriched at 50 °C on post-consumer PET (PET-50 °C) and virgin PLA granules (gPLA-50 °C), from 0 to 20 days after inoculum. (**B**) CFU counting of cultures enriched at 37 °C on post-consumer PET (PET-37 °C), virgin PLA granules (gPLA-37 °C), and post-consumer PLA cups (cPLA-37 °C), from 0 to 20 days after inoculum. (**C**) OD_600nm_ measurement of the enrichment cultures after the second medium replacement (i.e., 20th day).

Given the heterogeneity in colony morphologies and growth rates of the microbial inoculum, during the first 10 days, it was only possible to fragmentarily monitor microbial growth, especially for cultures incubated at 50 °C (Figure 1A), which included microbes that quickly spread all over the surface of the solid medium used for CFU counting (Figure S1B, upper and middle row). Nonetheless, a tenfold increase in cell concentration was observed within a timeframe of 8 days in the gPLA-50 culture. After the first spent medium exchange (i.e., 10 days post-inoculum), microbial biomass reached 10^8^ CFU/mL in both microbiomes enriched at 50 °C and was stably maintained until a decline in biomass content (i.e., 20th day). Hence, microbial proliferation was observed at 50 °C despite the initial inoculum being from a wastewater treatment plant operating under mesophilic conditions (25–35 °C). Regarding the cultures enriched at 37 °C (Figure 1B), the growth profiles exhibited an overlapping trend. A tenfold increase in biomass concentration was observed after the first 3 days of cultivation, followed by a slight decline until the 10th day. After the first medium replacement, microbial biomass increased from 10^5^ to about 10^9^–10^10^ CFU/mL over 3 days of cultivation, after which a threefold decrease was observed for cPLA-37 °C. After the second medium replacement (i.e., 20th day post-inoculum), microbial proliferation was monitored by means of OD_600nm_ measurements (Figure 1C). The latter displayed an increase in the cell density despite reaching different microbial biomass yields (of OD_600nm_/mL) within 10 days of analysis (i.e., from the 20th to the 30th day post-inoculum). Cell proliferation was confirmed in all cultures after the second medium replacements. As the following spent medium exchanges did not alter the medium composition (see Section 5.4), further growth analyses were not considered necessary. The latest spent medium exchange (i.e., 70th day post-inoculum) resulted in the exclusive presence of PET or PLA as the carbon source.

**Figure 2:**
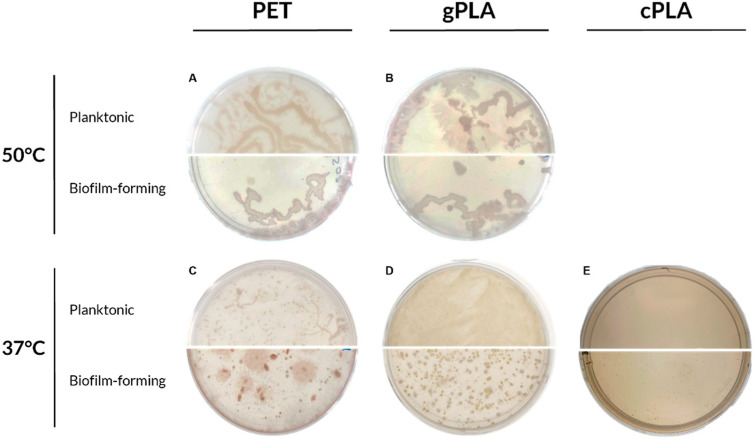
Microbiomes proliferated on plastic-containing solid media. The enriched microbiomes were seeded on plastic-containing agar plates after 100 days of cultivation. (**A**) PET-50 °C; (**B**) gPLA-50 °C; (**C**) PET-37 °C; (**D**) gPLA-37 °C; (**E**) cPLA-37 °C. Planktonic and biofilm-forming communities are shown in the upper and lower half of the agar plate, respectively.

To prepare glycerol stocks, following an additional 30-day enrichment period, microbiomes were cultivated onto solid medium containing PET and PLA emulsions (Figure 2).

Both planktonic and biofilm-forming microbiomes enriched at 50 °C reported limited morphological differences (Figure 2A,B). Additionally, a modest morphological variation was noted between PET-50 °C (Figure 2A) and gPLA-50 °C (Figure 2B) microbiomes. Despite the enrichment on different plastic polymers, the higher temperature promoted the selection of morphologically similar swarming bacteria. Conversely, microbiomes enriched on PET or PLA at mesophilic conditions (Figure 2C–E) exhibited a greater degree of morphological diversity. For instance, in the PET-37 °C consortium (Figure 2C), an evident morphological difference is appreciable within the planktonic and biofilm-forming communities, showing red fusiform colonies embedded in the solid medium, as well as punctiform and expanse colonies. This can be attributed to the source of the inoculum, which originated from a WWTP operating under mesophilic conditions; thus, at 37 °C, more members of the initial inoculum could survive to selection and proliferate. Moreover, the microbiomes enriched on the three different polymers exhibited distinctive morphological characteristics, indicating that the plastic polymer exerted a greater influence on selection than temperature.

### 2.2. Characterization of Plastic Polymers

Chemical characterization of polymers was performed by nuclear magnetic resonance (^1^H-NMR), gel permeation chromatography (GPC), and elemental analysis (CHNS); selected samples were also analysed by thermal analysis (TGA-DSC) and InfraRed (ATR FT-IR) spectroscopy. Non-incubated plastic samples (BLK) were analysed first, followed by the abiotic controls (AC) and the samples collected from the enrichment cultures (EC) at different temperatures (37 °C and 50 °C). NMR analysis of PET samples clearly showed that microbiomes enriched on PET did not have any hydrolytic activity towards this kind of plastic polymer. Indeed, the different PET samples showed comparable number average molecular weight (Mn) values, as can be judged by the ratio of repeating unit and end group integrals in the NMR spectra (Figure S2). Elemental analysis indicated that some slight modification of PET may occur in terms of C/H atomic ratios, but mainly at 50 °C (Table S1). This prompted us to abandon further investigations on PET.

On the contrary, PLA was efficiently degraded by the consortia (Table 1). NMR measurements confirmed a remarkable decrease in Mn for PLA samples incubated with the microbiomes (Figure S2). This behaviour was confirmed by GPC analyses, which provided further information on molecular weight distribution: weight average molecular weight (Mw) and polydispersity index (*D*) (Figure S3). In detail, post-consumer PLA cups (Table 1, cPLA, entries 1 to 3) showed only a low molecular weight decrease upon abiotic incubation at 37 °C (Mn reduction from 95,000 g mol^−1^ to 66,100 g mol^−1^) while being fully degraded after incubation with the microbiome at the same temperature (Mn drops to 1000 g mol^−1^). A similar behaviour was observed for the virgin PLA granules (Table 1, gPLA, entries 4 to 6) at 37 °C: abiotic incubation led to partial degradation (pristine sample Mn = 118,100 g mol^−1^, after abiotic incubation Mn = 49,400 g mol^−1^) while treatment with the microbiome at the same temperature led to complete depolymerization, yielding low molecular weight oligomers (Mn = 600 g mol^−1^). Finally, the same analysis carried out on the microbiome enriched at 50 °C (Table 1; gPLA, entries 7–8) clearly showed that evident depolymerization took place in the abiotic control, while exposure to the microbiome led to a further molecular weight decrease. NMR and GPC findings were also confirmed by CHNS analysis, especially for PLA granules (Table 1; gPLA, entries 4 to 8). In fact, a general decrease of the C/H atomic ratio, which is consistent with PLA depolymerization (Figure S4), was observed because of both the temperature (i.e., following abiotic incubation) and especially because of the exposure to the microbiomes (i.e., following biotic incubation) (Table 1).

Data obtained by NMR, GPC and CHNS on both PET and PLA are also in good agreement with those obtained by thermal analysis. In particular, while the thermogravimetric (TG, DTG) and the differential scanning calorimetry (DSC) curves of PET_EC-37°C_ are extremely similar to those of PET_BLK_, suggesting almost no degradation, a significant shift versus lower temperatures occurred for all endothermic peaks in DTG and DSC curves when gPLA_EC-37°C_ and gPLA_BLK_ are compared (Figure S5). This confirms that, following biotic incubation, gPLA showed a lower thermal stability, due to both chemical decomposition (i.e., depolymerization; Figure S4) and physical degradation (i.e., increase of the specific surface area). Similar indications were obtained on the same selected samples by ATR-IR spectroscopy, showing almost overlapping spectra for PET_EC-37°C_ and PET_BLK_, and the occurrence of new absorbance peaks (e.g., 1290 cm^−1^; C-O stretching), the disappearance of early ones (e.g., 1260 cm^−1^; C-H wag) and a variation of the relative intensity of others (1755 cm^−1^; C=O stretching) in gPLA_EC-37°C_ vs. gPLA_BLK_ (Figure S6).

### 2.3. Microbiomes and Sequence Analysis

**Figure 3:**
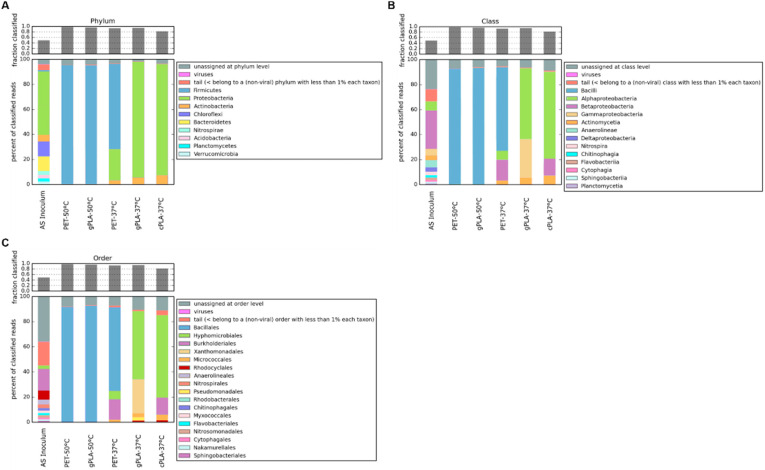
Bacterial taxonomic classification. The relative abundances of microbial phyla (**A**), classes (**B**), and orders (**C**) are shown for each sample. Relative abundances are expressed as the percentage of the total reads, classified against the NCBI nr database.

Metagenomic analysis was conducted to elucidate changes in the composition of microbial communities and to evaluate the presence of microorganisms and protein coding sequences associated with polyester degradation. Genomic DNA from both the initial AS and the enriched microbiomes was subjected to sequencing, yielding an average read count of approximately 30 million base pairs per sample. The raw reads in the AS, as well as in the enriched microbiomes, were assigned almost entirely to *Bacteria*. A total of nine phyla were found in the AS sample (Figure 3A), with the predominance of *Proteobacteria*, accounting for 50% of all the taxa assigned. The less dominant phyla included *Bacteriodetes* (13%), *Chloroflexi* (12%), *Actinobacteria* (5%), and *Firmicutes* (1%), which is in accordance with other studies of AS microbial composition in the literature [35]. A discrepancy between the sample and the literature was observed regarding the proportion of reads assigned to *Archaea* and *Eukarya*. In reference data, this proportion is approximately 10%, while in our sample, it is reduced to ≈1%. This discrepancy could be due to differences in the sequencing depth. Regarding the samples enriched at 50 °C, it is evident that temperature exerted a significant influence on the selection process, rather than the polymer composition. This is evident also by looking to a similar total n° of ORFs and the similar type of enrichment (≈nine times) of putative PLA/PET polyester degrading enzymes in 50 °C samples treated with different types of polyesters, if compared to the reference AS left at 37 °C (Table 2). Indeed, the two microbiomes exhibited a shared taxonomic profile, characterized by the enrichment of the *Bacillales* order (Figure 3C). The latter contains strains characterized for the degradation of PET and predominantly PLA [36]. Specifically, 20% of the PLA-active enzymes (PLAse) in the PAZy [20] and PlasticDB [37] databases were retrieved from members of the *Bacillales* order.

At 37 °C, a higher microbial diversity was observed compared to the microbiomes enriched at 50 °C. At the phylum level (Figure 3A), consortia gPLA-37 °C and cPLA-37 °C showed a similar taxonomic profile, i.e., *Proteobacteria* (~90%) and *Actinobacteria* (~7%). However, despite *Actinobacteria* being the most populated phylum of PLA degraders, the most represented orders (i.e., *Pseudonocardiales* and *Streptosporangiales*) were not enriched in the microbiomes. This seems to be confirmed by the apparent lack of any enrichment of PET/PLA-degrading ORFs with respect to the reference condition (Table 2). In contrast, the *Micrococcales* order was found to be enriched in the microbiomes selected at 37 °C. Within the *Proteobacteria* phylum, different bacterial orders were enriched in the presence of the two types of PLA. The gPLA-37 °C microbiome exhibited a higher representation of *Gammaproteobacteria* (particularly of *Xanthomonadales* and *Pseudomonadales*), whereas the cPLA-37 °C microbiome includes a majority of *Betaproteobacteria* (especially of *Burkorderiales*) (Figure 3B). Indeed, several PLA-active microorganisms belong to these microbial orders, predominantly to *Pseudomonadales*.

Considering the PET-37 °C microbiome, the orders *Bacillales* (67%), *Burkorderiales* (16%), *Hyphomicrobiales* (7%), and *Micrococcales* (3%) were enriched (Figure 3C). Known PET-degrading microorganisms fall predominantly within the *Actinobacteria* (e.g., *Streptosporangiales*) and *Proteobacteria* (e.g., *Pseudomonadales*), from which the best-performing enzymes for PET degradation were discovered [18]. However, a few strains belonging to the *Bacillales* and *Burkorderiales* orders also showed in vivo PET degradation abilities [32,38].

## 3. Discussion

Plastic has become an essential material for many industrial sectors, including automotive, construction, textiles and packaging, and demand for it is continuously growing. Indeed, plastic production is projected to triple by 2060, with population growth and emerging economies identified as key drivers of this rise [14]. However, current policies are likely to result also in an increase in unsustainable end-of-life practices (e.g., landfill and mismanagement) with adverse effects on all ecosystems. Consequently, there is a compelling need for sustainable approaches to plastic waste management, aligning with a circular economy model. To this end, biocatalytic approaches have been investigated to prompt recycling and upcycling of plastic waste. This research is consistent with previous findings that microplastic-polluted industrial niches, such as WWTPs, may serve as a reservoir for polyester-degrading microorganisms and enzymes [39,40]. In this context, the present study investigates the polyester-degrading capabilities of a WWTP microbiome, after selective enrichments on PET and PLA polymers. The enrichments were conducted under mesophilic and mildly thermophilic conditions. Thermophilic bacteria have gained interest due to their ability to produce thermostable enzymes that are active near the glass transition temperature of their substrate (65–70 °C for PET in water, ≈60 °C for PLA in water). Near the T_g_, these enzymes benefit from higher polymer chain mobility, leading to a higher degradation rate [41].

Following a 100-day enrichment period, PET and PLA were retrieved from the cultures and subjected to chemical characterisation using NMR, GPC and elemental analysis. No evidence of PET degradation was observed in terms of Mn reduction under either enrichment temperature. This finding is consistent with the fact that post-consumer PET from water bottles is a semicrystalline polymer showing high recalcitrancy to microbial and enzymatic hydrolysis. To date, the most effective PET hydrolases have demonstrated the capacity to degrade only the accessible amorphous regions of semicrystalline polymers, resulting in overall low levels of degradation [18].

In contrast, PLA samples exhibited significant reductions in molecular weight, in comparison to the abiotic controls, as confirmed by NMR and GPC analyses. At 37 °C, post-consumer PLA cups and virgin PLA granules were reduced to low molecular weight oligomers, indicative of successful biodegradation by the enriched microbiomes. At 50 °C, the elevated temperature facilitated the disintegration of gPLA pellets over a 100-day incubation period, with a further decrease in molecular weight in the presence of the gPLA-50 °C microbiome. This enhanced depolymerization likely occurs because the temperature of enrichment approaches the T_g_ of gPLA, allowing microbial activity to more severely break down the plastic polymers. These findings are in accordance with the existing literature on the higher susceptibility of PLA to microbial attack compared to PET [42], and on the role of temperature in promoting PLA degradation [43]. Furthermore, they align with the fact that PLA decomposes in industrial composting settings (>60 °C) within 180 days [44]. The situation is completely different in municipal compost facilities or the natural environment where temperatures are lower, and PLA takes years to biodegrade. Indeed, the consortia enriched in this study may offer potential applications in municipal compost facilities, accelerating the degradation of PLA at lower temperatures. However, for broader in-situ applications, such as in landfills or in contaminated sites, it would be advantageous to supplement these consortia with additional isolates capable of degrading other plastic polymers, including polyethylene, polypropylene, and polystyrene [45,46]. Notably, these polymers represent approximately one-third of the polymers commonly found in activated sludge [34]. This could suggest that activated sludge microbiomes, when properly selected [47], could exhibit additional plastic-degrading capabilities beyond those observed and enriched in this study. Alternatively, ex situ remediation could involve the use of engineered microbial strains equipped with known plastic-degrading enzymes optimized for activity at host living conditions. Furthermore, engineering strains with improved biofilm formation capabilities could enhance microbial colonization on plastic surfaces, increasing degradation efficiency [48].

Metagenomic analysis highlighted significant temperature-dependent variations in the microbial community structure and enzymatic capability. At 50 °C, microbiomes displayed a convergence in taxonomic composition dominated by *Bacillales*, which are well-documented for the encoding of thermophilic PLA-degrading enzymes [37]. This aligns with the enrichment in gPLA-50 °C and PET-50 °C microbiomes of ORFs predicted as PET/PLA hydrolases. In contrast, at 37 °C, a more diverse microbial composition emerged, comprising *Proteobacteria* and *Actinobacteria* as dominant phyla along with *Firmicutes* in the PET-37 °C microbiome. Despite successful PLA degradation and the enrichment of potential PLA-active microorganisms, no sequence enrichment for putative PLA depolymerase was observed. However, most of the characterised PLA depolymerases demonstrate optimal activity at thermophilic conditions (i.e., between 50 °C and 70 °C [49,50]) while mesophilic enzymes tend to be more promiscuous and active at alkaline pH values [51,52,53], deviating from the neutral pH of the enrichment (pH 7.0). Moreover, using the sequence of these enzymes as a reference for the HMM search and the application of a restrictive similarity threshold may have resulted in the inability to observe any enrichment in divergent putative PLA depolymerases, even if biodegradation has occurred. Taken together, these observations might suggest the presence of novel mesophilic PLA depolymerases distinct from those already characterised in the literature.

Further investigations will focus on the functional characterization of the microbiomes and the identification of putative PLA depolymerase responsible for polymer hydrolysis.

## 4. Conclusions

This study confirms the potential of activated sludge microbiomes as reservoirs for plastic-degrading enzymes, particularly for PLA hydrolysis. Enrichment cultures demonstrated the adaptability of microbial consortia under mesophilic and thermophilic conditions, with significant PLA degradation measured through chemical analyses. Metagenomic analyses revealed temperature-dependent shifts in microbial community composition, with *Bacillales* dominating at moderate thermophilic conditions and diverse *Proteobacteria* and *Actinobacteria* thriving at mesophilic temperatures. In mesophilic conditions, the extensive PLA biodegradation together with the lack of enrichment of PLA-degrading enzymes suggests the presence of novel enzymes and/or degradation pathways in these populations. These enzymes could advance biotechnological applications for sustainable plastic waste management. Future work will focus on identifying and characterizing these biocatalysts to enhance plastic re- and up-cycling strategies.

## 5. Materials and Methods

### 5.1. Activated Sludge Characterization

The AS was collected from the municipal wastewater treatment plant (WWTP) of Acque Veronesi S.c.r.l. As reported in Bertanza et al., 2011 [54], the key operational data, with typical values, are as follows: influent water flow: 92,000 m^3^/day (under dry weather conditions); dissolved oxygen concentration in aeration tanks: 2.0–2.2 mg/L; total suspended solids concentration in biological reactors: 4.0–4.5 g TSS/L. Influent characteristics (post-screening and grit-oil removal) include the following: 450 mg COD/L, 200 mg BOD_5_/L, 240 mg TSS/L, 50 mg TKN/L, and 5 mg total phosphorus (P_tot_)/L. Effluent characteristics are as follows: 30 mg COD/L, 5 mg BOD_5_/L, 12 mg TSS/L, 6.5 mg TKN/L, 4 mg NH_4_⁺-N/L, 4 mg NO_3_^−^-N/L, less than 0.1 mg NO_2_^−^-N/L, and 1.3 mg P_tot_/L [54].

AS was characterized in terms of total solid (TS), total volatile solid (TVS), and ash content, according to Eaton et al. (1998) [55]. The results of the analysis are summarized in Table 3.

### 5.2. Media and Chemicals

The defined mineral medium used in this study to carry out microbial enrichment and selection was named plastic enrichment medium (PEM, pH 7.0). The medium composition was adapted from Kim et al. (2017) [29]. In brief, it contains the following components (*w*/*v*): 0.2% (NH_4_)_2_SO_4_, 1% Na_2_HPO_4_·2H_2_O, 0.5% NaH_2_PO_4_, 0.0062% NH_4_Cl, and 0.0026% KCl. Trace elements, vitamins, and FeSO_4_ solutions (Table S2) were separately prepared as concentrated stocks, filtered with sterile 0.22-μm cut-off polyethersulfone (PES) membranes (LLG labware), and then added to PEM after autoclavation to avoid mineral precipitations and vitamin inactivation. PEM was supplemented with 0.01% (*w*/*v*) yeast extract (YE) and 0.3% (*w*/*v*) sodium acetate (NaOAc), to prepare the PEM-AY medium containing organic carbon and nitrogen sources to sustain microbial growth at the beginning of the enrichments. When needed, culture media were supplemented with 1.5% (*w*/*v*) agar (Neofroxx, Einhausen, Germany) for microbial cultivation on solid media in 90-mm Petri dishes (Sarsted).

PET and PLA were obtained from post-consumer water bottles and cups (cPLA), respectively. Post-consumer PET and cPLA were cut into 1 cm^2^ pieces and sterilised by immersion in a 70% (*v*/*v*) ethanol solution for 2 h. Then, the plastic pieces were rinsed with sterile dH_2_O and dried aseptically. Virgin PLA granules (gPLA) were kindly provided by the company Corbion (Luminy^®^ LX175) and sterilized as described above for PET and cPLA.

### 5.3. Plastic-Containing Agar Plates

Plastic-containing agar plates were used to isolate biofilm-forming and planktonic microbiomes from the enrichment cultures. PET and cPLA were emulsified in agarised PEM as described in Charnock (2021) [56], with minor modifications. Briefly, PET from post-consumer water bottles or cPLA were cut into 0.25 cm^2^ pieces. PET and cPLA were dissolved in dimethylsulfoxide (DMSO; Honeywell, Charlotte, NC, USA) at a final concentration of 0.03 g/mL, at 180 °C and 100 °C respectively, under slight stirring for 10 min. The agarised PEM medium was autoclaved at 121 °C for 20 min and kept at 85 °C during the preparation of the plastic suspension. After the sterilization process, the medium was supplemented with 0.0045% (*v*/*v*) Tween-20 (Chemlab) and with 0.5 mL of the plastic solution. The latter was added dropwise with the use of a preheated glass Pasteur pipette under sonication (Bandelin SONOPULS, 40% amplitude) and slight stirring. The plastic-containing PEM medium was cast in Petri dishes and stored at 4 °C for up to a month.

### 5.4. Enrichment Cultures

The AS from a local WWTP was used as the microbial inoculum for the enrichment of plastic-active microorganisms in batch cultures containing PET, cPLA or gPLA. To avoid excessive dilution of the AS organic content (i.e., mainly microbes), 2 L of AS was centrifuged at 5000× *g* for 10 min and the pellet was resuspended in an equal volume of PEM-AY. Subsequently, 300 mL of this inoculum solution was added to five 1-L baffled Erlenmeyer flasks containing PET, cPLA or gPLA, as reported in Table 4.

**Figure 4:**
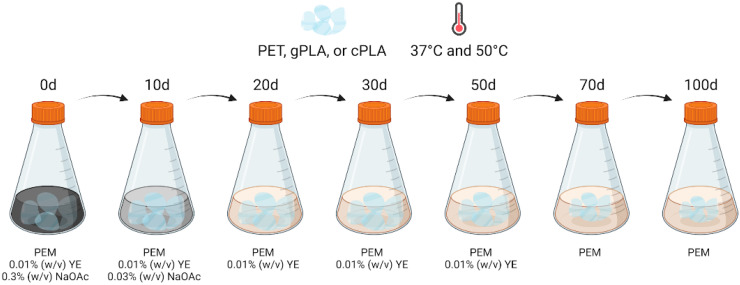
Schematic representation of medium exchange during enrichment. Plastic enrichment medium (PEM); yeast extract (YE); sodium acetate (NaOAc). Created in BioRender. Salini, A. (2024) BioRender.com/c10z513.

Abiotic controls were set up for each polymer by replacing the microbial inoculum with sterile PEM-AY supplemented with 0.05% (*w*/*v*) of sodium azide. The enrichment cultures, as well as the abiotic controls, were incubated at 37 °C and 50 °C under a constant shaking rate of 100 rpm, to promote the selection of mesophilic and mildly thermophilic microbial communities (i.e., microbiomes). Then, the nutrient content of the medium was gradually reduced over time, as described in Kim et al. (2017) [29], with minor modifications. The spent medium was aseptically removed from each flask using a serological pipette, and 300 mL of fresh medium was added (Figure 4).

Specifically, at the 10th day post-inoculation, the spent PEM-AY medium was replaced with PEM supplemented with 0.01% (*w*/*v*) yeast extract and 0.03% (*w*/*v*) NaOAc. Then, spent medium exchanges were performed on the 20th, 30th, and 50th day post-inoculation, using PEM containing only 0.01% (*w*/*v*) yeast extract to provide only minimum organic nitrogen and carbon sources. At the 70th day post-inoculation, the yeast extract was also omitted from the medium to leave PET, gPLA or cPLA as the only carbon sources left in the medium (Figure 4).

Lastly, enrichment cultures were sampled at the 100th day post-inoculation to isolate the microbes that had survived the selection on plastic. To do so, 10 mL of medium from each flask was centrifuged at 5000× *g* for 15 min to collect the planktonic cells. The cell pellets were resuspended in PEM, which was seeded on PET-, gPLA-, or cPLA-containing agar plates (see Section 5.3) and incubated at their respective temperature of enrichment until a mat of cells was visible on the plate surface. Additionally, a few PET and PLA pellets were collected from every culture to isolate the biofilm-forming microbial community. First, the plastic pieces were gently washed three times with a sterile solution of 0.9% (*w*/*v*) NaCl to remove all the unattached cells. Then, they were vortexed in 5 mL of 0.9% (*w*/*v*) NaCl for 3 min to recover the biofilm-forming cells [57], which were seeded on PET-, gPLA-, or cPLA-containing agar plates. The latter were incubated at their respective temperature of enrichment upon the appearance of a mat of cells. All microbiomes were collected from the solid medium using a sterile solution of 0.9% (*w*/*v*) NaCl and stored as glycerol stocks at −80 °C.

#### 5.4.1. Microbial Biomass Measurements

Colony-forming unit (CFU) counting was carried out on the agarised Luria-Bertani (LB) medium after the first medium exchange to monitor cell growth. In brief, 10-fold serial dilutions of the cultures were prepared using sterile 0.9% (*w*/*v*) NaCl. Then, 100 µL of each dilution was seeded onto agarised LB and incubated overnight at the temperature of enrichment. Plates on which a countable number of microbial colonies appeared were used for CFU counting. After the second medium exchange, cell proliferation was monitored with the use of a spectrophotometer via OD_600_ measurements until 30 days after inoculum.

#### 5.4.2. NMR and GPC Analyses

Nuclear magnetic resonance (NMR) spectroscopy and gel permeation chromatography (GPC) analyses were carried out on pieces of PET, gPLA and cPLA collected from the following: (i) the enrichment cultures (EC) and named as PET_EC-37°C_, gPLA_EC-37°C_, cPLA_EC-37°C_, PET_EC-50°C_, and gPLA_EC-50°C_ as well as from (ii) the abiotic controls (AC), set up to evaluate the effect on the plastics of the sole incubation, and labelled as PET_AC-37°C_, gPLA_AC-37°C_, cPLA_AC-37°C_, PET_AC-50°C_, and gPLA_AC-50°C_. Additionally, non-incubated PET, gPLA and cPLA were processed as blank (BLK) controls, and referred to as PET_BLK_, gPLA_BLK_, and cPLA_BLK_. Prior to analyses, the plastic pieces were sterilised by immersion in a 70% (*v*/*v*) ethanol solution for 2 h. Then, they were immersed in 2% (*w*/*v*) sodium dodecyl sulphate (SDS) solution for 24 h with gentle shaking, to remove the cells from the surface of the polymer. After rinsing with dH_2_0, the plastic pellets were dried overnight at 37 °C. ^1^H (400 MHz) NMR spectra were recorded in CDCl_3_ or CDCl_3_:TFA = 1:1 at room temperature. The chemical shifts in ^1^H spectra were referenced to tetramethylsilane (TMS). NMR spectra are reported in Figure S2.

Gel permeation chromatography (GPC) was conducted using a system consisting of a Jasco PU-4180 pump operating at a flow rate of 0.4 mL/min, two PLgel miniMIXED-D columns in series, a Jasco CO-4060 column oven, a 20 µL injector, a Jasco RI-4030 refractive index detector, and a Jasco UV-4075 multi-channel UV-Vis detector. The column oven was maintained at 30 °C, and HPLC-grade chloroform was used as the eluent. Calibration was performed using polystyrene standards. GPC chromatograms are reported in Figure S3.

#### 5.4.3. Elemental Analysis

The concentration of carbon (C), nitrogen (N), hydrogen (H) and sulphur (S) in PLA and PET samples was determined by flash combustion using a CHNS analyser (Vario Macro cube, Elementar, Germany). Oxygen was determined by difference (100-(C+N+H+S)). Sulphanilamide (Elementar Analysensysteme GmbH, Langenselbold, Germany) was used as a standard, while alfalfa organic analytical standard (OAS) (B2273, Elemental Microanalysis Limited, Okehampton, UK) was used as a reference material. The accuracy (QA/QC) was >98% for all elements. Elemental analysis data are reported in Table S1.

#### 5.4.4. Thermal and InfraRed Analyses

Selected PLA and PET samples were analysed using a thermogravimetric analyser (TG) coupled with differential scanning calorimetry (DSC) (TGA-DSC 3+, Mettler Toledo, Greifensee, Switzerland) and equipped with STARe Software V16.20 (Mettler, Greifensee, Switzerland). Samples were placed in an alumina crucible and heated from 20 to 600 °C at 5 °C min^−1^ under an atmosphere of nitrogen at a flow rate of 50 mL min^−1^.

Attenuated total reflectance Fourier transform infrared (ATR FT-IR) spectra were recorded on the same selected samples over the range of 4000–400 cm^−1^ using a Nicolet™ iS50 FTIR Spectrometer with an ATR accessory (Thermo Fisher Scientific, Carlsbad, CA, USA) and equipped with Nicolet Omnic 9.13.1224 software, at a spectral resolution of 4 cm^−1^ and with 64 scans for each acquisition.

### 5.5. Microbial Consortia Analysis

#### 5.5.1. DNA Extraction and Quality Analyses

DNA extraction was performed using the DNeasy PowerSoil Pro Kit (QIAGEN), following the manufacturer’s instructions. Regarding the isolated consortia, the Wizard SV Genomic DNA Purification System (Promega) was used to purify the genomic DNA, following the manufacturer’s instructions. Prior to genomic DNA extraction, the isolated consortia were cultured on solid media containing the same plastic polymer used for their enrichment (see Section 5.3) and then incubated at the specific enrichment temperature to allow microbial growth. Later, cells were collected with sterile 0.9% (*w*/*v*) sodium chloride and pelleted at 5000× *g* for 5 min. The cell pellets were washed twice with sterile 0.9% (*w*/*v*) sodium chloride, to remove impurities that could interfere with genomic DNA extraction.

The purity of DNA samples was qualitatively analysed using a NanoDrop (Thermo Fisher Scientific, Carlsbad, CA, USA). The DNA concentrations were estimated using an Invitrogen Qubit 4 Fluorometer (Thermo Fisher Scientific, Carlsbad, CA, USA), following the manufacturer’s instructions. Genomic DNA was analysed with gel electrophoresis on a 0.8% (*w*/*v*) agarose (marca) gel in Tris-acetate-EDTA (TAE) buffer supplemented with 1X EuroSafe Nucleic Acid Staining solution (20,000X, Clinisicence). The samples were mixed with loading buffer (0.25% *v*/*v* glycerol, 0.01% *w*/*v* bromophenol blue, 62.5 mM Tris-Hcl buffer pH 6.8). The electrophoretic run was conducted at 60 V for 2 h and the gel was imaged through Chemidoc (Biorad, Hercules, CA, USA). The genomic DNA was sequenced by IGA Technology Services, on NovaSeq6000 (Illumina, San Diego, CA, USA) in paired-end mode (150 base pairs).

#### 5.5.2. Sequence Analyses

Metagenomic data analysis was performed using the KBASE platform [58]. Sequencing reads underwent preprocessing, involving the removal of low-quality bases and the exclusion of human contamination, using Trimmomatic v0.36 [59]. The reads were taxonomically classified using Kaiju v1.7.3 [60]. Metagenome assemblies were independently carried out for each sample using the MetaSPAdes app (v3.15.3) [61]. The open reading frames were identified using prodigal v.2.6.3 [62] and annotated as esterase putatively capable of hydrolysing PET or PLA by sequence similarity to distinct hidden Markov models (HMMs), generated with hmmbuild from HMMer v.3.4 [63] from separate alignments, each obtained through MAFFT v.7.525 [64] (“einsi” and “MAFFT-DASH” options); alignments were made of sequences of reference PET and PLA hydrolases from distinct serin hydrolase families (each family consisting of characterized sequences with >20% global pairwise identity). Reference PET/PLA hydrolases were selected from the PlasticDB database (https://plasticdb.org/database, accessed on 16 February 2024) [37] and the recently characterized PAM enzyme [65] was added to this list. A max restrictive e-value of e^−30^ was allowed for the annotations.

## Figures and Tables

**Table 1:** Chemical characterization of PLA polymers. Characterization of cPLA and gPLA by Nuclear Magnetic Resonance (^1^H NMR), Gel Permeation Chromatography (GPC) *^a^* and elemental analysis (CHNS).

Entry	Sample	M_n(NMR)_ *^b^* (g mol^−1^)	M_n(GPC)_ *^c^* (g mol^−1^)	M_w(GPC)_ *^c^* (g mol^−1^)	*D ^c^*	C/H *^d^*
1	cPLA_BLK_	n.d.	95,000	177,400	1.9	0.68
2	cPLA_AC-37°C_	n.d.	66,100	124,500	1.9	0.67
3	cPLA_EC-37°C_	1300	1100	5000	4.5	0.69
4	gPLA_BLK_	n.d.	118,100	215,200	1.8	0.77
5	gPLA_AC-37°C_	55,300	49,400	117,000	2.4	0.76
6	gPLA_EC-37°C_	950	600	1000	1.7	0.70
7	gPLA_AC-50°C_	1000	600	900	1.5	0.74
8	gPLA_EC-50°C_	800	n.d.	n.d.	n.d.	0.71

*^a^* See Section 5 for sample preparation and analysis details. *^b^* Determined by ^1^H-NMR (CDCl_3_). *^c^* Determined by GPC. *^d^* Atomic ratio determined by elemental analysis.

**Table 2:** Statistics on metagenomic open reading frames (ORFs) annotated with PET/PLA hydrolase activity.

Sample Name	n° ORFs		Relative PLA Hydrolase Content with Respect to AS INOCULUM
	PLA Hydrolase Annotated	Total	
AS inoculum	39	186,302	1
PET-37 °C	10	29,560	1.62
PET-50 °C	8	4269	8.95
cPLA-37 °C	3	22,820	0.63
gPLA-37 °C	8	40,131	0.95
gPLA-50 °C	8	4255	8.98

**Table 3:** TS and TVS characterization of the AS. The average and the standard deviation of three independent measures are shown.

Activated Sludge	
TS (mg/L)	8.68 ± 0.09
TVS (mg/L)	5.85 ± 0.09
Ash (mg/L)	2.84 ± 0.10

**Table 4:** Experimental setup of the five enrichment batch cultures.

Sample Name	Polymer Type	Polymer Concentration	Incubation Temperature
PET-37 °C	Post-consumer PET bottles	10% (*w*/*v*)	37 °C
gPLA-37 °C	Virgin PLA granules	33% (*w*/*v*)	37 °C
cPLA-37 °C	Post-consumer PLA cups	10% (*w*/*v*)	37 °C
PET-50 °C	Post-consumer PET bottles	10% (*w*/*v*)	50 °C
gPLA-50 °C	Virgin PLA granules	33% (*w*/*v*)	50 °C

## Data Availability

Sequencing data have been submitted to the SRA database, BioProject ID PRJNA1074543.

## Supplementary Materials

The following supporting information can be downloaded at: https://www.mdpi.com/article/10.3390/ijms252312747/s1.
