# Supplementary material for: Plastic-Degrading Microbial Consortia from a Wastewater Treatment Plant
Source: Int J Mol Sci. 2024 Nov 27;25(23):12747. doi: 10.3390/ijms252312747 (PMC11641126; doi:10.3390/ijms252312747)
Supplement: Supplementary file 1 [file ijms-25-12747-s001.zip › ijms-3326804-Supplementary Materials.pdf]

Supplementary Materials

Plastic-degrading microbial consortia from a Wastewater Treatment Plant

Andrea Salini[1], Luca Zuliani[1], Paolo Matteo Gonnelli[1], Marco Orlando[2], Andrea Odoardo[3], Daniele Ragno[3], Martina Aulitto[4], Claudio Zaccone[5] and Salvatore Fusco[1,*]

[1] Biochemistry and Industrial Biotechnology (BIB) Laboratory, Department of Biotechnology, University of Verona, 37134 Verona, Italy; salvatore.fusco@univr.it
[2] Department of Biotechnology and Biosciences, University of Milano Bicocca, Milano, 20126 Italy; marco.orlando@unimib.it
[3] Department of Chemical, Pharmaceutical and Agricultural Sciences, University of Ferrara, 44121 Ferrara,
Italy; daniele.ragno@unife.it
[4] Department of Biology, University of Naples Federico II; martina.aulitto@unina.it
[5] Lab of Soil and Biomass Chemistry, Department of Biotechnology, University of Verona, 37134 Verona, Italy; claudio.zaccone@univr.it
* Correspondence: salvatore.fusco@univr.it

Figure S1. Enrichment of microbial consortia. A) Appearance of the enrichment culture before the second medium replacement (i.e., 20th day post inoculum). B) CFU counting of the microbial consortia on LB supplemented with 1.5% (w/v) agar.

A **B**

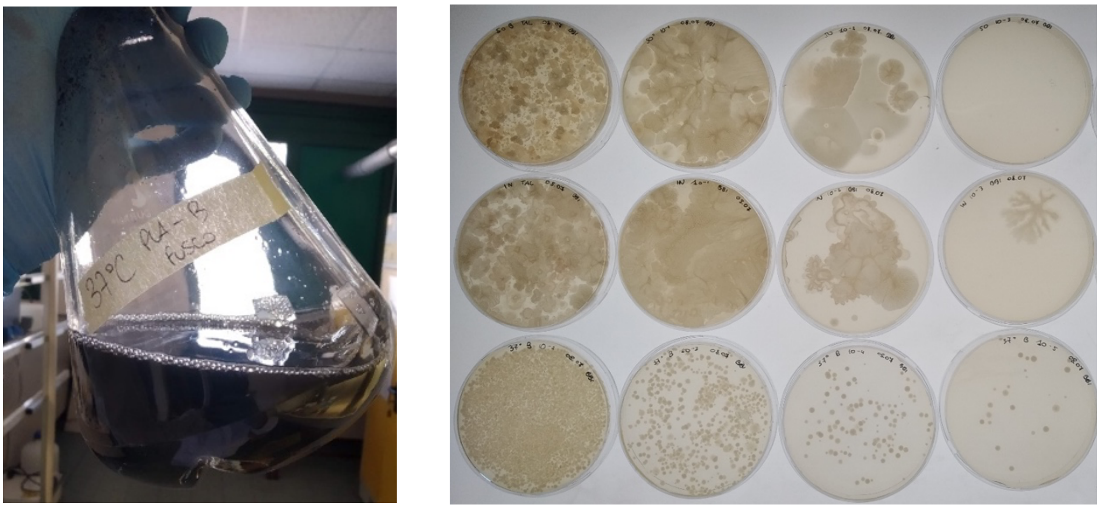

Figure S2. ^{1}H (400 MHz) NMR spectra ($CDCl_3$) carried out on plastic polymers (cPLA and gPLA), and ($CDCl_3$:TFA = 1 : 1) on PET. A) cPLA$_{BLK}$; B) cPLA$_{AC\text{-}37°C}$; C) cPLA$_{EC\text{-}37°C}$; D) gPLA$_{BLK}$; E) gPLA$_{AC\text{-}37°C}$; F) gPLA$_{EC\text{-}37°C}$; G) gPLA$_{AC\text{-}50°C}$; H) gPLA$_{EC\text{-}50°C}$; I) PET$_{BLK}$; J) PET$_{AC\text{-}37°C}$; K) PET$_{EC\text{-}37°C}$; L) PET$_{AC\text{-}50°C}$; M) PET$_{EC\text{-}50°C}$.

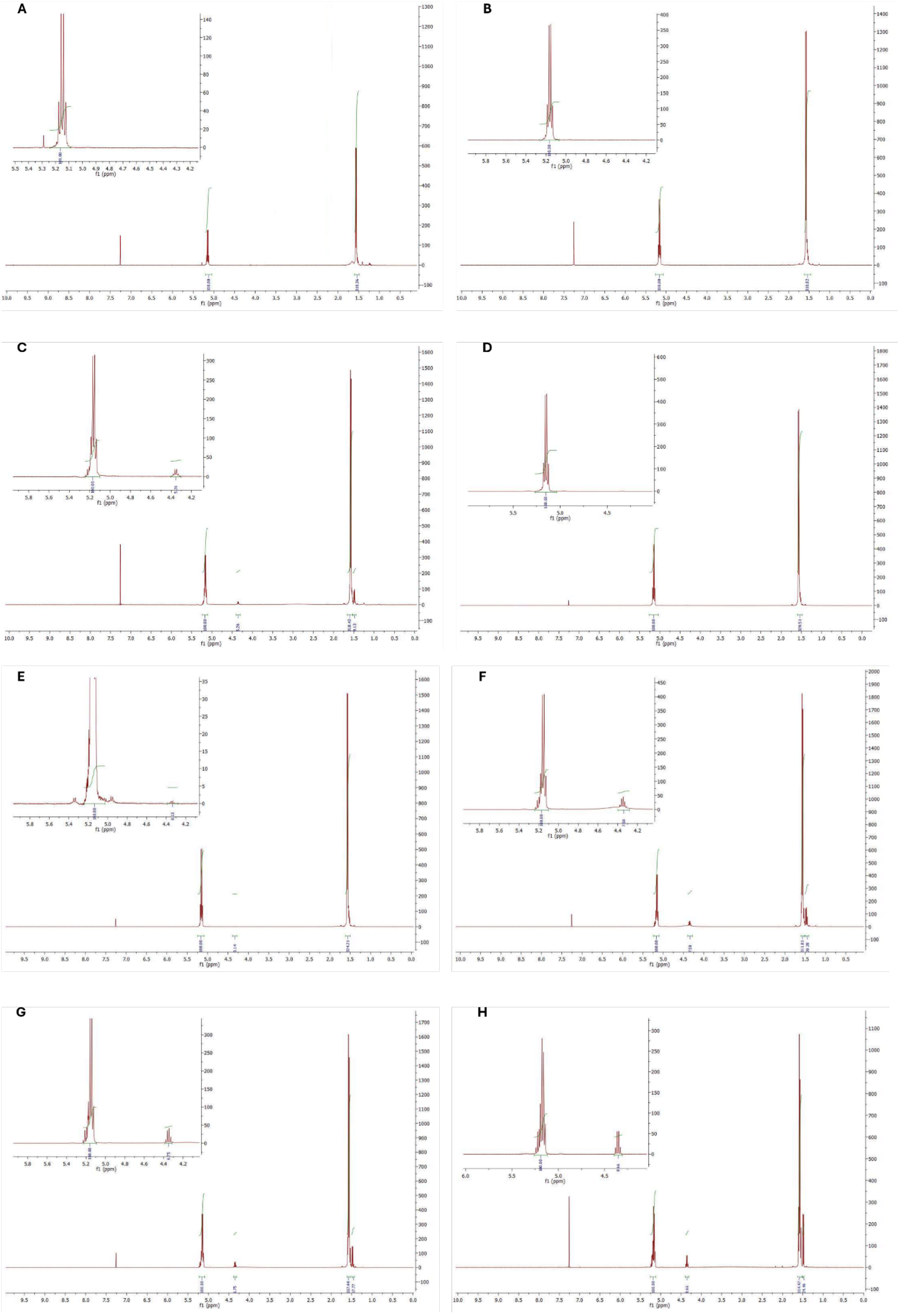

I

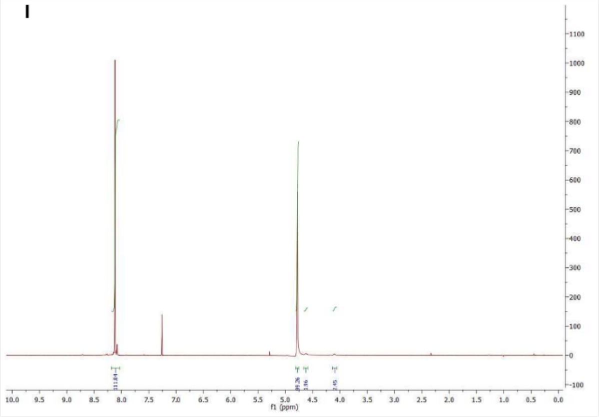

J

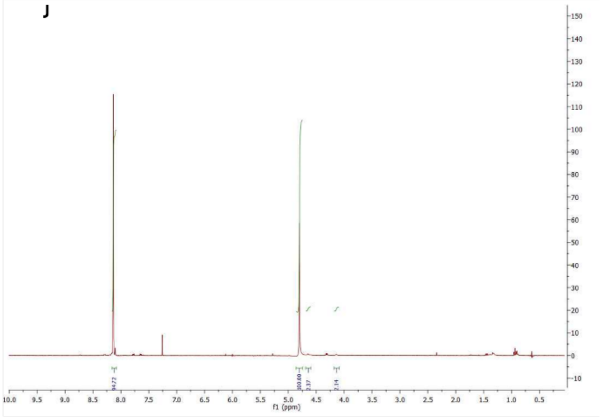

K

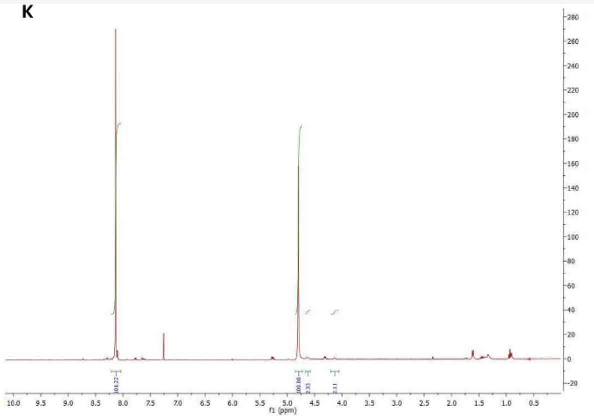

L

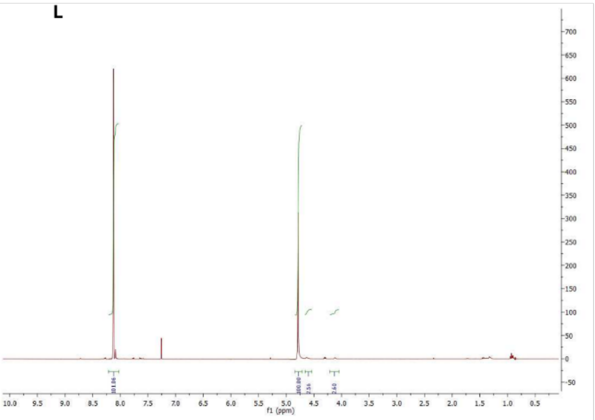

M

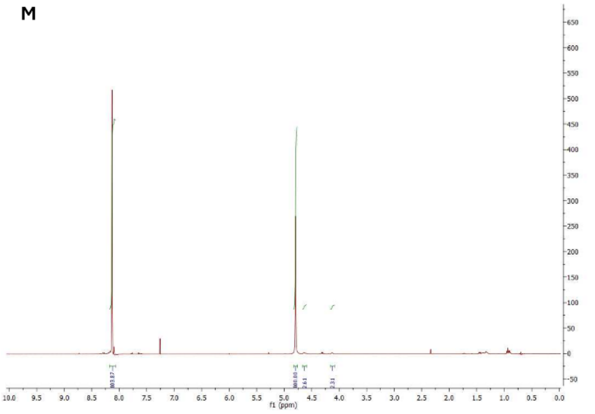

Figure S3. GPC chromatograms of plastic polymers. A) $cPLA_{BLK}$; B) $cPLA_{AC\text{-}37°C}$; C) $cPLA_{EC\text{-}37°C}$; D) $gPLA_{BLK}$; E) $gPLA_{AC\text{-}37°C}$; F) $gPLA_{EC\text{-}37°C}$; G) $gPLA_{EC\text{-}37°C}$. For sample $gPLA_{EC\text{-}50°C}$, the molecular weight was under the lower limit of detection (Mn < 500 g*mol^{-1}).

A

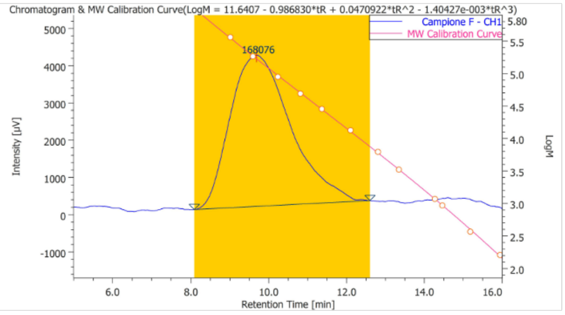

#	Range	CH	tR	Mn	Mw	Mw/Mn	Range Value	Percentage
1	8.0800 – 12.6000	1	9.6833	95044	177452	1.8670	439804	100.00

B

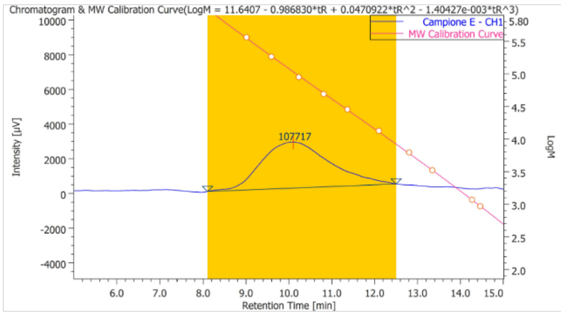

#	Range	CH	tR	Mn	Mw	Mw/Mn	Range Value	Percentage
1	8.1000 – 12.5000	1	10.0967	66149	124473	1.8817	296334	100.00

C

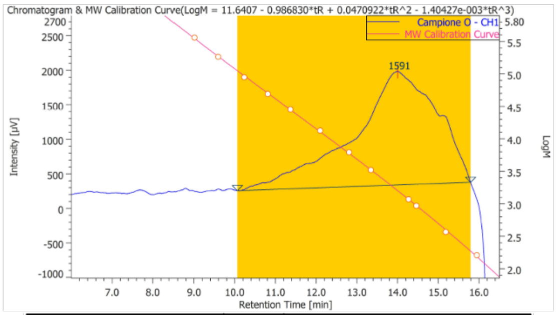

#	Range	CH	tR	Mn	Mw	Mw/Mn	Range Value	Percentage
1	10.0600 – 15.7900	1	14.0000	1128	5037	4.4636	235088	100.00

D

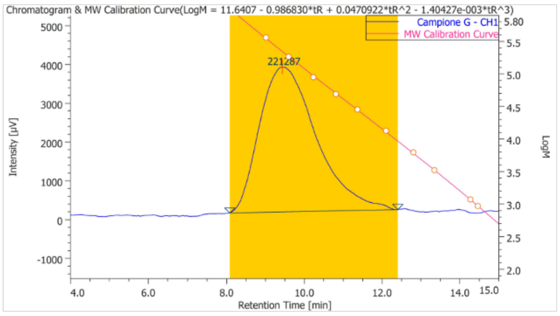

#	Range	CH	tR	Mn	Mw	Mw/Mn	Range Value	Percentage
1	8.0800 – 12.4000	1	9.4300	118128	215235	1.8221	379504	100.00

E

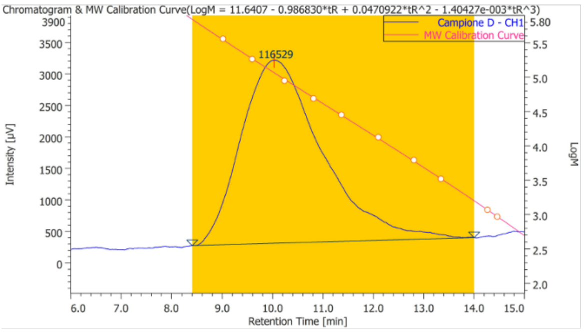

#	Range	CH	tR	Mn	Mw	Mw/Mn	Range Value	Percentage
1	8.4000 – 14.0000	1	10.0233	49453	116960	2.3651	324866	100.00

F

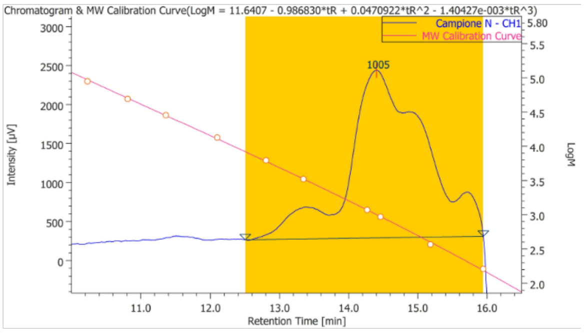

#	Range	CH	tR	Mn	Mw	Mw/Mn	Range Value	Percentage
1	12.5000 – 15.9400	1	14.4000	636	1077	1.6937	165874	100.00

G

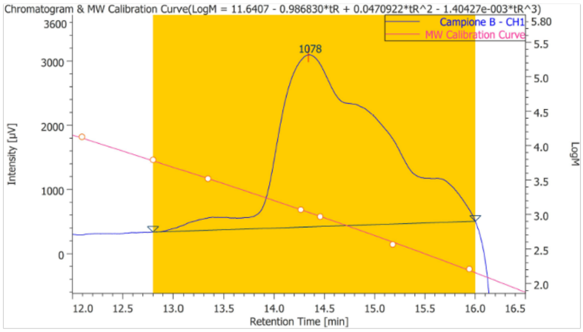

#	Range	CH	tR	Mn	Mw	Mw/Mn	Range Value	Percentage
1	12.8000 – 16.0000	1	14.3400	602	904	1.5022	189215	100.00

Figure S4. Schematic representation of the enzymatic hydrolysis of PLA. Upon hydrolysis the C/H ratio decreases from a theoretical maximum of 0.75 (repeating unit) to 0.50 (lactic acid monomer). Image created in BioRender.com

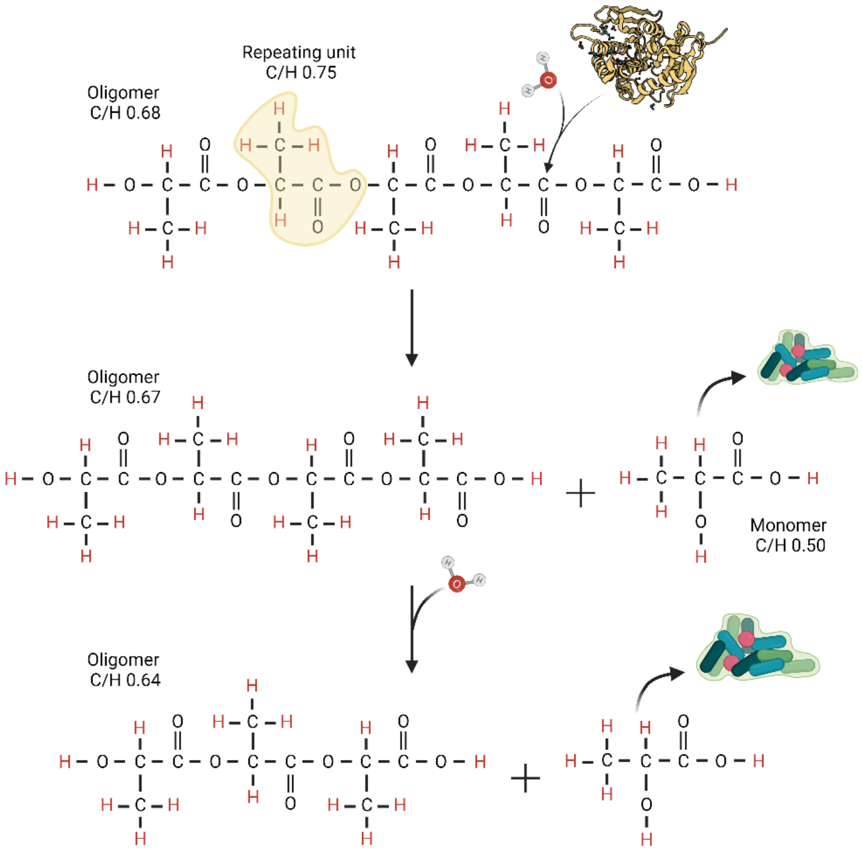

Figure S5: Thermal analysis on selected PET and PLA samples. A) Thermogravimetric (TG) curves, B) first derivative of TG curves (DTG), and C) differential scanning calorimetry (DSC) curves of PET_{BLK} *vs.* $PET_{EC\text{-}37°C}$ (in red) and of $gPLA_{BLK}$ *vs.* $gPLA_{EC\text{-}37°C}$ (in grey).

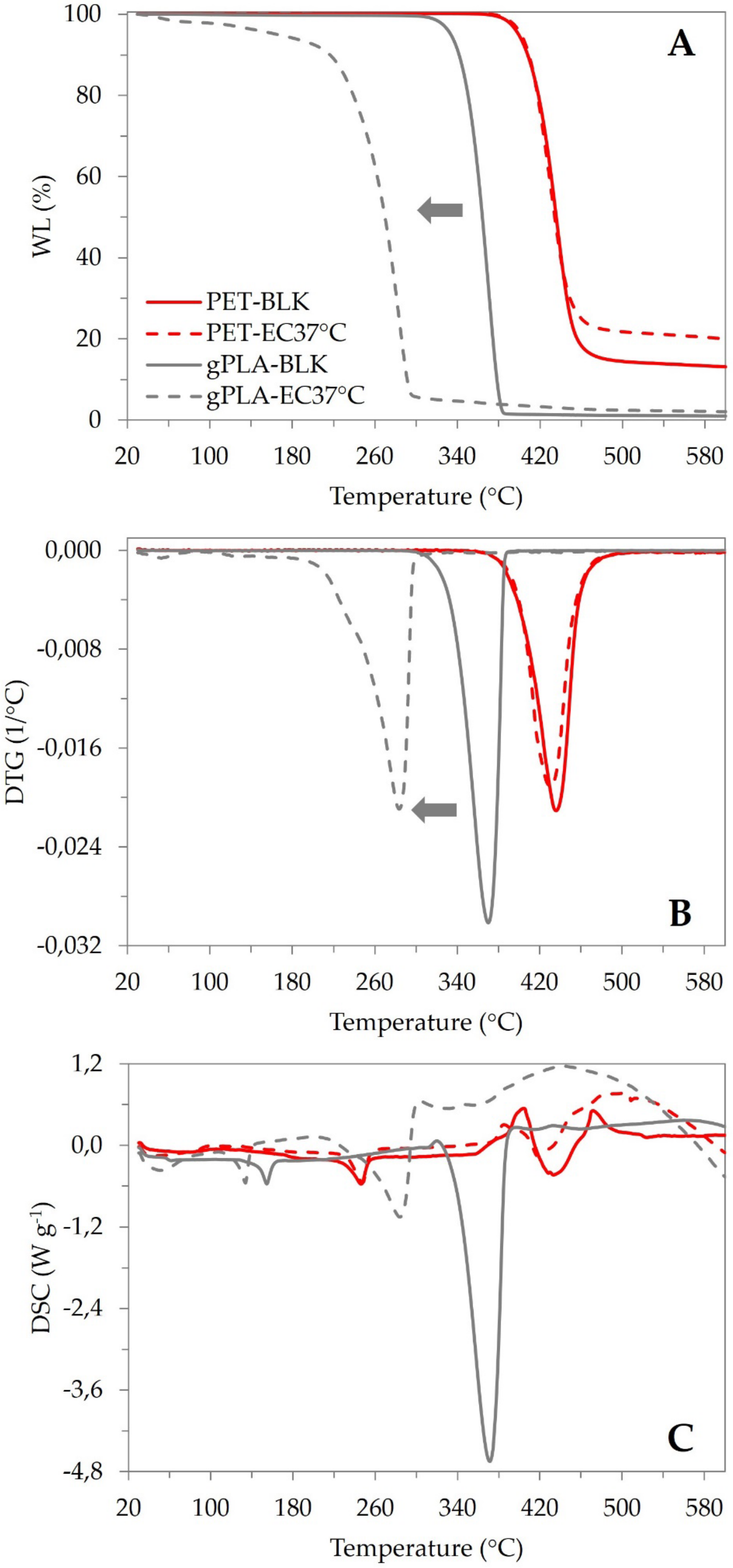

Figure S6: Attenuated total reflectance Fourier transform infrared (ATR FT-IR) spectra of selected PET and PLA samples. On the top, PET_{BLK} *vs.* $PET_{EC\text{-}37°C}$; on the bottom, $gPLA_{BLK}$ *vs.* $gPLA_{EC\text{-}37°C}$.

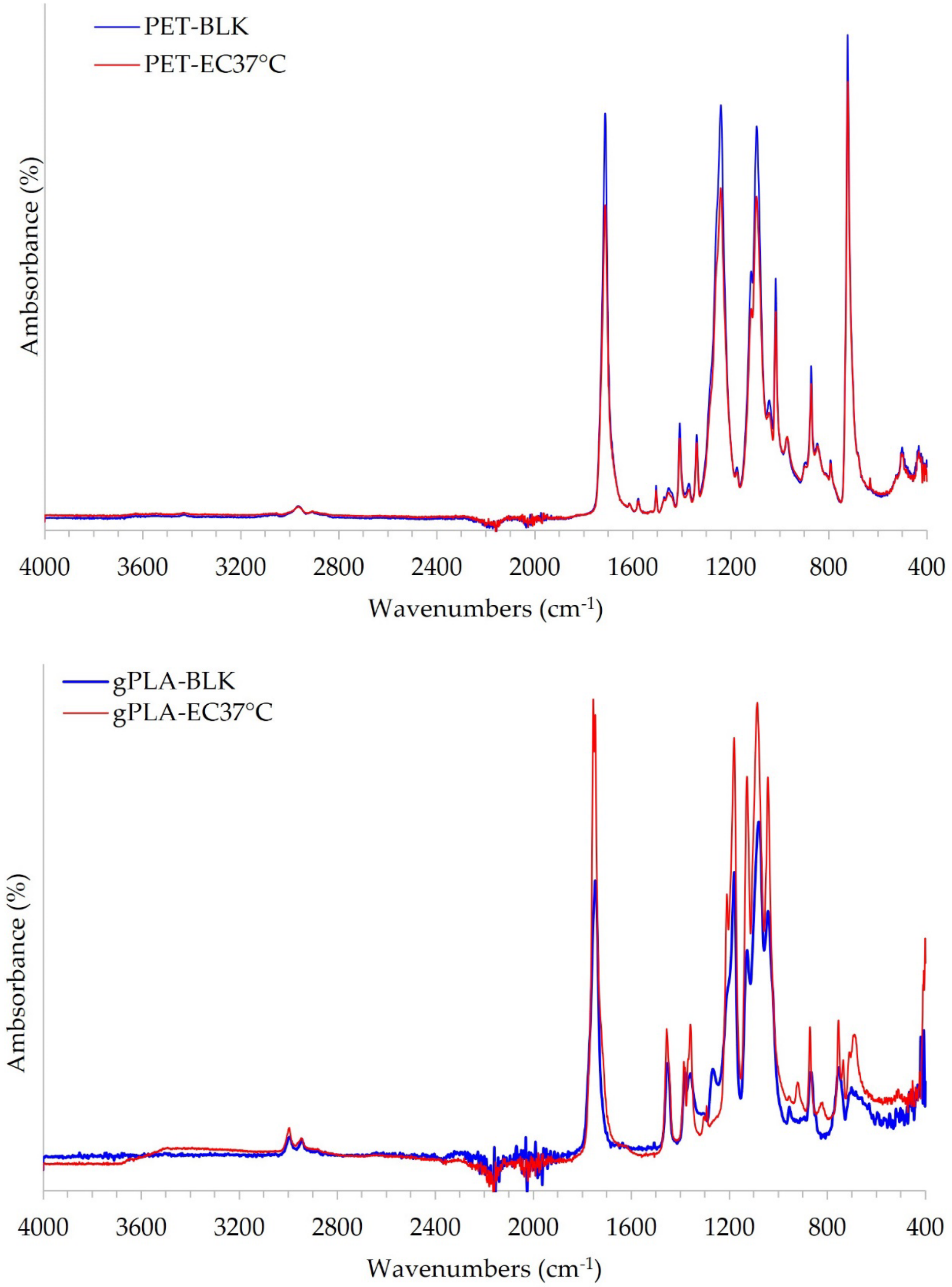

Table S1. Elemental analysis. The table shows the percentage of N, C, H, S, and O (*Determined by difference: O = 100-(C+N+H+S)) present in the polymers collected from the enrichment (EC), the abiotic controls (AC) and the not incubated controls (BLK). LOQ for N = 0.01%; LOQ for S = 0.05%.

	N [%]	C [%]	H [%]	S [%]	O [%] *	C/H atomic ratio
$cPLA_{BLK}$	<LOQ	50.21	6.06	0.366	43.35	0.68
$cPLA_{AC-37°C}$	<LOQ	49.98	6.20	0.276	43.53	0.67
$cPLA_{EC-37°C}$	<LOQ	48.98	5.83	0.091	45.06	0.69
$gPLA_{BLK}$	<LOQ	50.02	5.39	<LOQ	44.54	0.77
$gPLA_{AC-37°C}$	<LOQ	49.97	5.42	0.071	44.53	0.76
$gPLA_{EC-37°C}$	<LOQ	48.80	5.79	<LOQ	45.36	0.70
$gPLA_{AC-50°C}$	<LOQ	49.36	5.54	0.215	44.88	0.74
$gPLA_{EC-50°C}$	<LOQ	48.41	5.64	0.097	45.85	0.71
PET_{BLK}	<LOQ	62.44	4.70	0.129	32.74	1.10
$PET_{AC-37°C}$	<LOQ	62.41	4.28	0.245	33.05	1.20
$PET_{EC-37°C}$	<LOQ	62.58	4.31	<LOQ	33.03	1.20
$PET_{AC-50°C}$	<LOQ	62.57	3.94	0.451	33.03	1.31
$PET_{EC-50°C}$	<LOQ	62.53	4.30	<LOQ	33.08	1.20

Table S2. Trace elements, vitamins and $FeSO_4$ solutions composition. Each stock solution was diluted to a working concentration of 1X for PEM preparation.

Trace elements solution (1000X)	% (w/v)
$MgSO_4 \cdot 7H_2O$	1
$CuSO_4 \cdot 5H_2O$	0.1
$MnSO_4 \cdot 5H_2O$	0.1
$ZnSO_4 \cdot 7H_2O$	0.1

Vitamin solution (2000X)	% (w/v)
Thiamine-HCl	1
Niacin	0.1
p-aminobenzoic acid	0.1
Pyridoxal-HCl	0.1
Pantothenate	0.01
Biotin	0.005
Vitamin B12	0.05

$FeSO_4$ solution in 0.5M HCl	% w/v
$FeSO_4 \cdot 7H_2O$	1
